# Prevalence and characteristics of HTLV-associated uveitis in patients from Bahia, an endemic area for HTLV − 1 in Brazil

**DOI:** 10.1186/s12985-023-02135-7

**Published:** 2023-08-21

**Authors:** Daniele Piai Ozores, Regina Rathsam Pinheiro, Ney Boa-Sorte, Maurício Campos e Silva Dias, Raianne Silva Lima, Thessika Hialla Almeida Araújo, Bernardo Galvão-Castro, Maria Fernanda Rios Grassi

**Affiliations:** 1 Escola Bahiana de Medicina e Saúde Pública, Salvador, BA Brazil; 2 Hospital Humberto Castro Lima, Instituto Brasileiro de Oftalmologia e Prevenção a Cegueira, Salvador, BA Brazil; 3 Instituto Gonçalo Moniz-Fundação Oswaldo Cruz, Salvador, BA Brazil; 4 HCOE – Hospital de Olhos, Feira de Santana, BA Brazil

**Keywords:** HTLV-1 uveitis (HAU), HAU uveitis types, Endemic area, Prevalence

## Abstract

**Background:**

HTLV-1-associated uveitis (HAU) is an inflammatory reaction of the choroid, retina, optic nerve and vitreous that can lead to vision impairment. The worldwide prevalence of HAU varies widely.

**Objective:**

To determine the prevalence of HAU in patients from Salvador, Bahia-Brazil, and describe uveitis type and associated symptoms.

**Methods:**

Cross-sectional analytical study to determine the prevalence of uveitis in HTLV-1-infected patients recruited in Bahia, Brazil, a region considered endemic for HTLV-1. Patients were enrolled at a local reference center for HTLV (infected) and at an outpatient ophthalmology clinic (noninfected group). All patients were examined by the same ophthalmologist following a single protocol. Prevalence ratios (PR) were calculated.

**Results:**

A total of 168 consecutively examined HTLV-1-infected patients and 410 noninfected patients (randomly selected) were included. Females predominated (82.1%) in the HTLV-1-infected group (versus 64.4% in the uninfected group) (p < 0.001). The mean age of infected and uninfected patients was 53.2 and 62.8 years, respectively (p < 0.001). The prevalence of uveitis in HTLV-1^+^ and HTLV-1^−^ patients was 7.14% and 0.73%, respectively (PR = 9.76; 95CI%:2.79–34.15; p < 0.01). Bilateral intermediate uveitis, associated with symptoms including visual disturbances and floaters, was most commonly identified in the HTLV-1-infected patients, whereas unilateral anterior uveitis, in association with symptoms such as blurring and ocular pain, was more common in the uninfected group.

**Conclusion:**

The prevalence of uveitis in patients with HTLV-1 was markedly higher than in uninfected subjects. HAU patients were mostly asymptomatic and exhibited bilateral presentation, with uveitis more frequently localized in the intermediate chamber.

## Introduction

Human lymphotropic virus type 1 or human T leukemia virus (HTLV-1) was the first human retrovirus to be isolated and associated with disease [1]. It is estimated that 5 to 10 million people are infected worldwide, primarily in Japan, the Caribbean, parts of the African continent, and South America [2]. In Brazil, the northeastern state of Bahia is considered an important endemic area for HTLV-1 infection, with approximately 130,000 infected persons [3].

HTLV-1 is the causative agent of diseases such as adult T-cell leukemia lymphoma, HTLV-1-associated myelopathy/tropical spastic paraparesis (HAM/TSP), infective dermatitis, and HTLV-associated uveitis (HAU) [4–9].

Uveitis is an inflammation of the choroid, retina, optic nerve and vitreous that can lead to visual impairment or blindness [10–12]. In addition to systemic/autoimmune diseases and idiopathic causes [10, 13], infectious diseases, such as toxoplasmosis, toxocariasis, herpes simplex, herpes zoster, cytomegalovirus and tuberculosis, may also induce uveitis and should be investigated [14]. The diagnosis of uveitis requires slit lamp biomicroscopy and retinal mapping to detect the presence of an inflammatory process.

In Japan, studies have estimated the prevalence of HTLV-1 in patients with idiopathic uveitis [15, 16]. However, few studies have attempted to determine the prevalence of HAU worldwide, with reported rates ranging from 1.6% in Argentina to 14.5% in Martinique [17, 18]. In Brazil, four studies have described HAU in 1.9–2.85% of patients [19–22]. However, studies on HAU prevalence only partially consider relevant ophthalmologic findings and do not perform differential diagnosis of uveitis; moreover, few studies included a control group not infected with HTLV-1 ^18,19,22^.

The present study endeavored to determine the prevalence of HAU in the city of Salvador, the capital of the state of Bahia, an endemic area for HTLV-1 in Brazil, as well as to describe the types of uveitis and associated symptoms identified in both HTLV-1-infected patients and uninfected subjects.

## Materials and methods

### Study design and population

The present cross-sectional, controlled study aimed to determine the prevalence of uveitis in HTLV-1-infected patients. The study was conducted between March 2019 to June 2021 at the HTLV Integrative and Multidisciplinary Center of the Bahia School of Medicine and Public Health (CHTLV/EBMSP) [23] in Salvador, and at the HCOE Ophthalmology Outpatient Clinic (Hospital de Olhos) in Feira de Santana, both neighboring municipalities in the state of Bahia.

### Inclusion and exclusion criteria

Patients infected with HTLV-1 (serological diagnosis: ELISA confirmed by Western Blot) were consecutively enrolled at CHTLV. HTLV-1 patients diagnosed with acute (IgM-positive) toxoplasmosis, toxocariasis, rubella or cytomegalovirus infection, or those with a previous diagnosis of syphilis, HIV, hepatitis B or C, tuberculosis, Crohn’s disease, ulcerative colitis, ankylosing spondylitis, Bechet’s disease, sympathetic ophthalmia, Harada’s disease or sarcoidosis were excluded. The control group of individuals not infected with HTLV-1 consisted of patients randomly selected (using the randomize.org program) by assigning an alphanumeric sequence to medical records.

The present study protocol was approved by the EBMSP Institutional Research Board; all participating patients provided signed informed consent or waived consent, as appropriate.

### Sample calculation

To calculate the sample size, we assumed a uveitis prevalence of ~ 6% in HTLV-1-infected patients [23] compared to 1% in the general noninfected population [11, 12, 24]. Thus, considering a confidence level of 95%, a power of 80% and a ratio of noninfected to infected patients of 3:1, the minimum sample size necessary was 118 HTLV-1-infected patients and 351 noninfected patients [25].

### Diagnosis of HTLV-1 infection

Anti-HTLV-1 antibodies were detected by ELISA and confirmed by Western blot assay. Patients infected with HTLV-1 were evaluated for the presence of myelopathy, and HAM/TSP was diagnosed using criteria established by WHO [8].

### Ophthalmologic examination

All ophthalmologic examinations were performed by an ophthalmologist (DPO) specialized in the diagnosis of uveitis. Ophthalmologic signs and symptoms were assessed through the comprehensive examination of both eyes: Measurement of visual acuity using Snellen charts, applanation tonometry, refractometry, biomicroscopy of the anterior and posterior eye chambers, retinal mapping, and tear film evaluation using tear film breakup time (BUT), Rose Bengal Staining 0.1%, and Schirmer I test. The diagnosis of keratoconjunctivitis sicca (KCS) was based on the presence of symptoms, along with positivity on at least two of the three latter tests, as described elsewhere [26].

The diagnosis of uveitis was based on a detailed ophthalmologic examination of the anterior, intermediate and posterior portions of the eye to detect the presence of an inflammatory reaction. Anatomic classifications were based on the International Uveitis Study Group, which designates uveitis into anterior, intermediate, posterior and panuveitis classifications [10].

Anterior and posterior biomicroscopy (performed via slit lamp) was employed to evaluate the presence or absence of anterior chamber reaction, flare, and keratic precipitates as indicators of anterior uveitis, as well as to analyze the cellularity of the anterior vitreous [10]. Anterior chamber response was assessed by determining the number of cells in the anterior chamber and quantified from 0 to 4 crosses, with 0 indicating < 1 cell observed per field, 0.5 + from 1 to 5 cells per field, 1 + from 6 to 15 cells per field, 2 + from 16 to 25 cells per field, 3 + from 26 to 50 cells per field, and 4 + over 50 cells per field. Opacities were graded from 0 to 4+, with 0 indicating no opacity, 1 + weak, 2 + moderate (iris and lens can be clearly visible), 3 + marked (iris and lens can be visualized but with opacities), 4 + severe (fibrin in aqueous humor).

Indirect binocular ophthalmoscopy was performed using an indirect binocular ophthalmoscope and a 20-diopter loupe after pupil dilation to assess the presence of intermediate and posterior uveitis. The presence of vitreous cellularity, snowballs, snowbanks, vasculitis, papillitis, retinitis, retinochoroiditis, exudates, and cystoid macular edema were noted. Vitreous cellularity was classified as 0 to 5 crosses, with 0 representing no opacities, 0.5 + representing trace cells, 1 + representing minimal opacities with clearly visible posterior pole, 2 + representing moderate opacities with mild posterior pole opacities, 3 + representing moderate opacities with severe posterior pole opacities, 4 + representing severe opacities with difficulty in posterior pole visibility, and 5 + representing severe opacities with no posterior pole visibility.

### Statistical analysis

Results were reported as proportions for categorical variables and as means ± standard deviation (SD) for continuous variables. Shapiro-Wilk test was used to test for normality. Statistical tests included the Student t test or Mann-Whitney non-parametric test for mean comparison of independent variables and the Pearson chi-square test or Fisher’s exact test for comparisons between proportions of ocular disease and sociodemographic data between HTLV-1-infected and uninfected patients. Differences were considered statistically significant when p < 0.05. We used Stata for Mac®, version 13.0.

Results were expressed as proportions for categorical variables and as means ± standard deviation (SD) for continuous variables. The Shapiro-Wilk test was used to test normality. To compare the means of age between the HTLV-1 and Control groups, the nonparametric Mann-Whitney test was used after no normality was established. Pearson’s chi-square test or Fisher’s exact test was used when indicated for comparisons between proportions of ocular disease and sociodemographic data between HTLV-1-infected and uninfected patients. Differences were considered statistically significant when p < 0.05. All data were analyzed using STATA v13.0.

## Results

The population studied consisted of 168 individuals infected with HTLV-1 and 410 uninfected patients who served as controls. The proportion of women in the HTLV-1-infected group (82.1%) was higher than in the uninfected group (64.4%), p < 0.001. The mean age of infected and uninfected patients was 53.2 ± 12.5 and 62.8 ± 14.5 years, respectively (p < 0.001).

The prevalence of uveitis was significantly higher in HTLV-1 infected patients than in uninfected subjects [7.14% (12 of 168) versus 0.73% (3 of 410); PR = 9.76%, CI95% (2.79–34.15) p < 0.001], (Table 1). Regarding the cause of uveitis, no other associations were found in the HTLV-1 patients, whereas in two subjects of the uninfected group, tuberculosis and herpes simplex were each associated with this disease. A third uninfected subject was diagnosed with uveitis, but without a clear etiological association.

**Table 1:** Prevalence of uveitis in HTLV-1-infected patients and uninfected controls

	HTLV-1 group N = 168	Control group N = 410
**Prevalence of uveitis (%)**	7.14	0.73
**Uveitis associated with HTLV**	12	0
**Uveitis associated with other diseases**	0	2*
**Undetermined uveitis**	0	1

*Tuberculosis and Herpes simplex

Bilateral uveitis was present in 66% (8 of 12) of HTLV-1-infected patients, whereas no individuals with bilateral uveitis were identified in the uninfected control group. Intermediate uveitis was the most common form identified in the HTLV-1-infected group (58.3%; 7 of 12), followed by anterior uveitis (25%; 3 of 12) and pan uveitis (16.7%; 2 of 12). In contrast, no cases of intermediate uveitis were observed in the uninfected controls. However, anterior uveitis was seen in 2 of 3 patients (66.6%) and pan uveitis in 1 of 3 (33.3%) (Table 2).

Regarding the main ocular complaints of patients diagnosed with uveitis, 50% (6 of 12) of HTLV-1-infected patients reported visual disturbances and floaters, whereas the other six patients (50%) were asymptomatic. In the uninfected group, all three patients reported symptoms of blurring and ocular pain.

**Table 2:** Characteristics of HTLV-1-associated uveitis and uveitis in uninfected individuals

Uveitis	HTLV-1 group N = 12	Control group N = 3
**Localization**		
Anterior	3	2
Intermediate	7	0
Posterior	0	0
Pan-uveitis	2	1
**Laterality**		
Bilateral	8	0
Unilateral	4	3
**Symptoms**		
Floaters	6	0
Visual blurring	6	3
Ocular pain	0	3
Asymptomatic	6	0

**Fig. 1:**
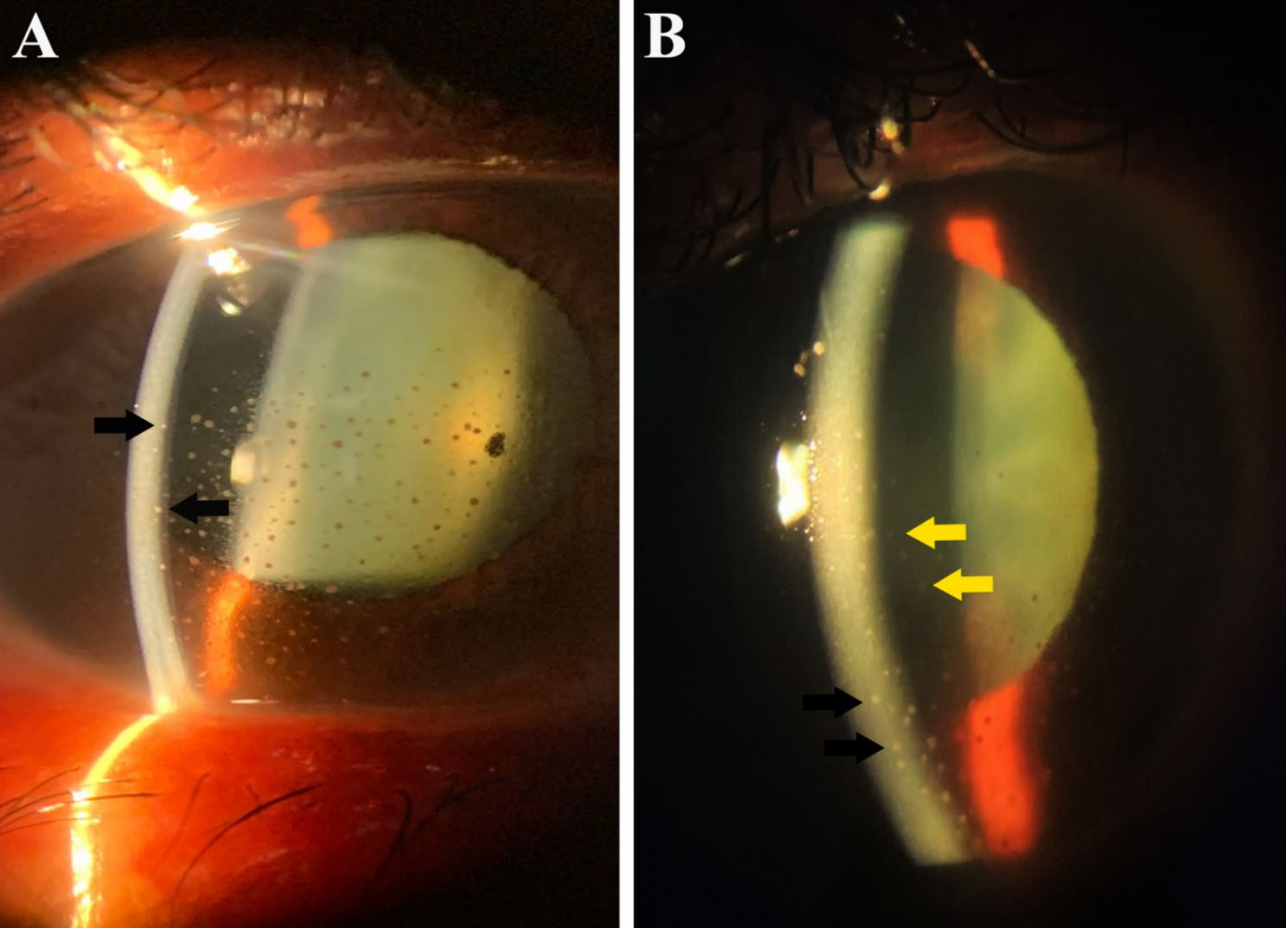
Endothelial keratic precipitates (black arrow) and anterior chamber reaction (yellow arrows) and in HTLV-1 patients (anterior slit-lamp biomicroscopic examination). Patients 11 and 1, respectively

**Fig. 2:**
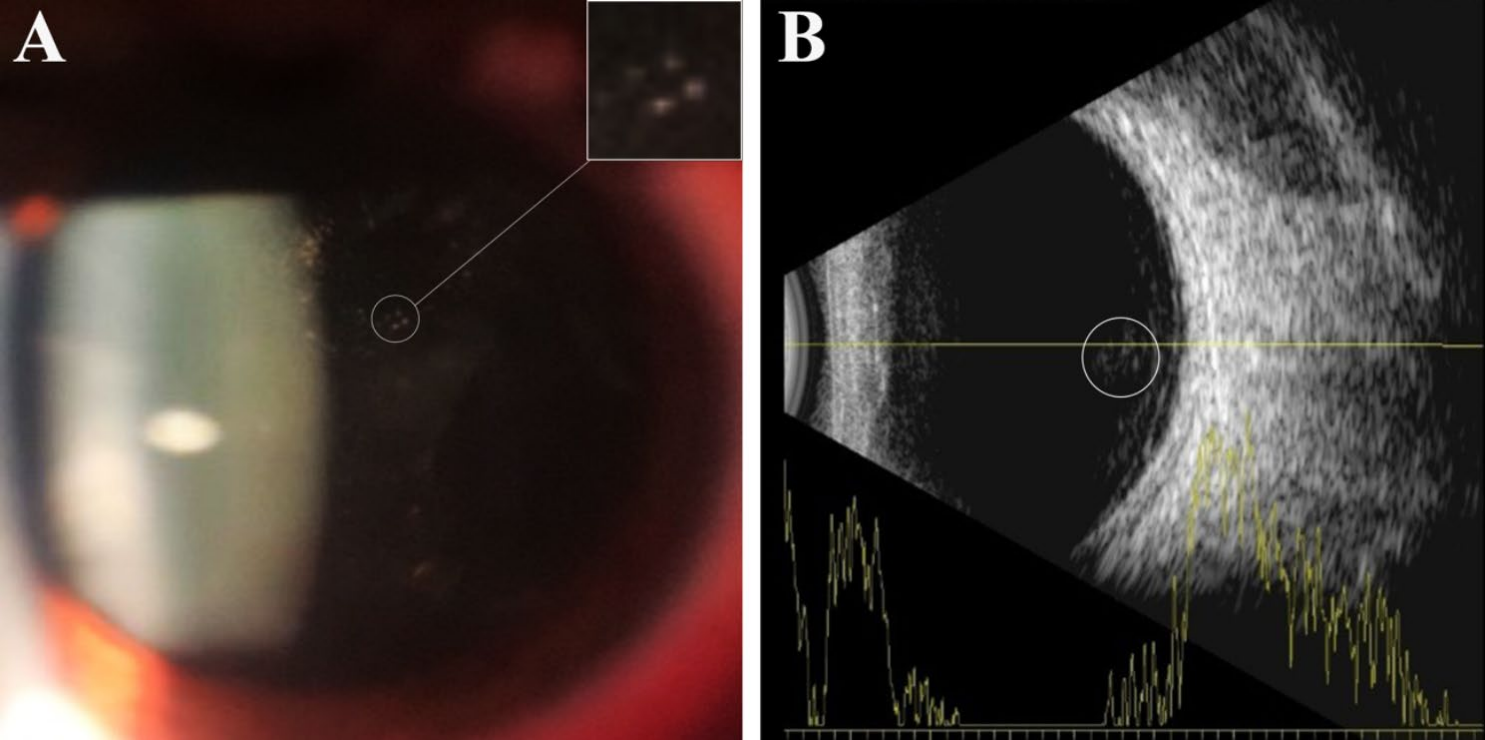
Anterior slit-lamp biomicroscopic examination (**A**) and ocular ultrasound (**B**) showing vitreous cells characteristic of active intermediate uveitis in an HTLV-1 patient (patient 3)

**Fig. 3:**
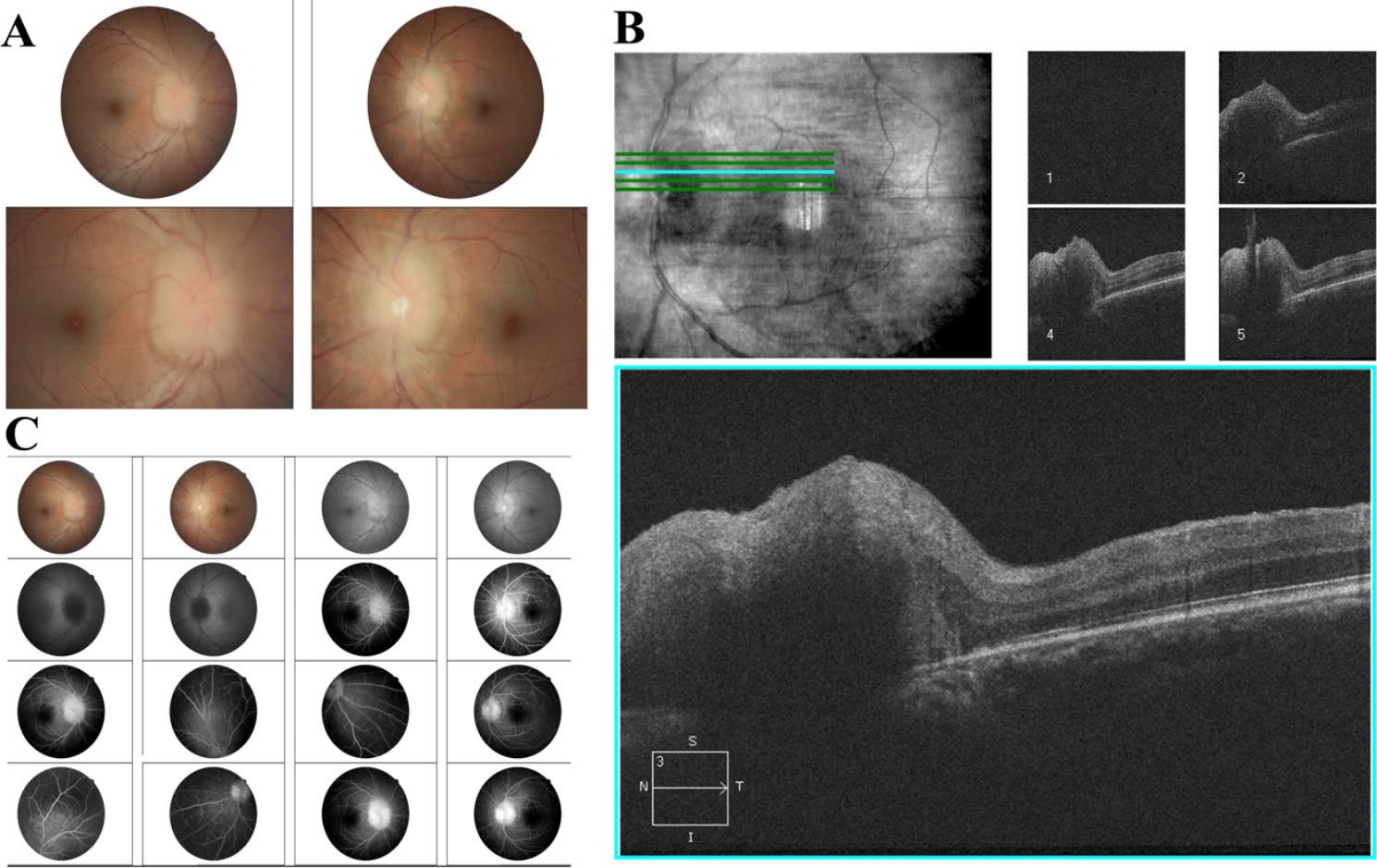
Papilledema in an HTLV-1 patient in retinography (**A**), optical coherence tomography (**B**) and fluorescein angiography (**C**). Patient 2

**Fig. 4:**
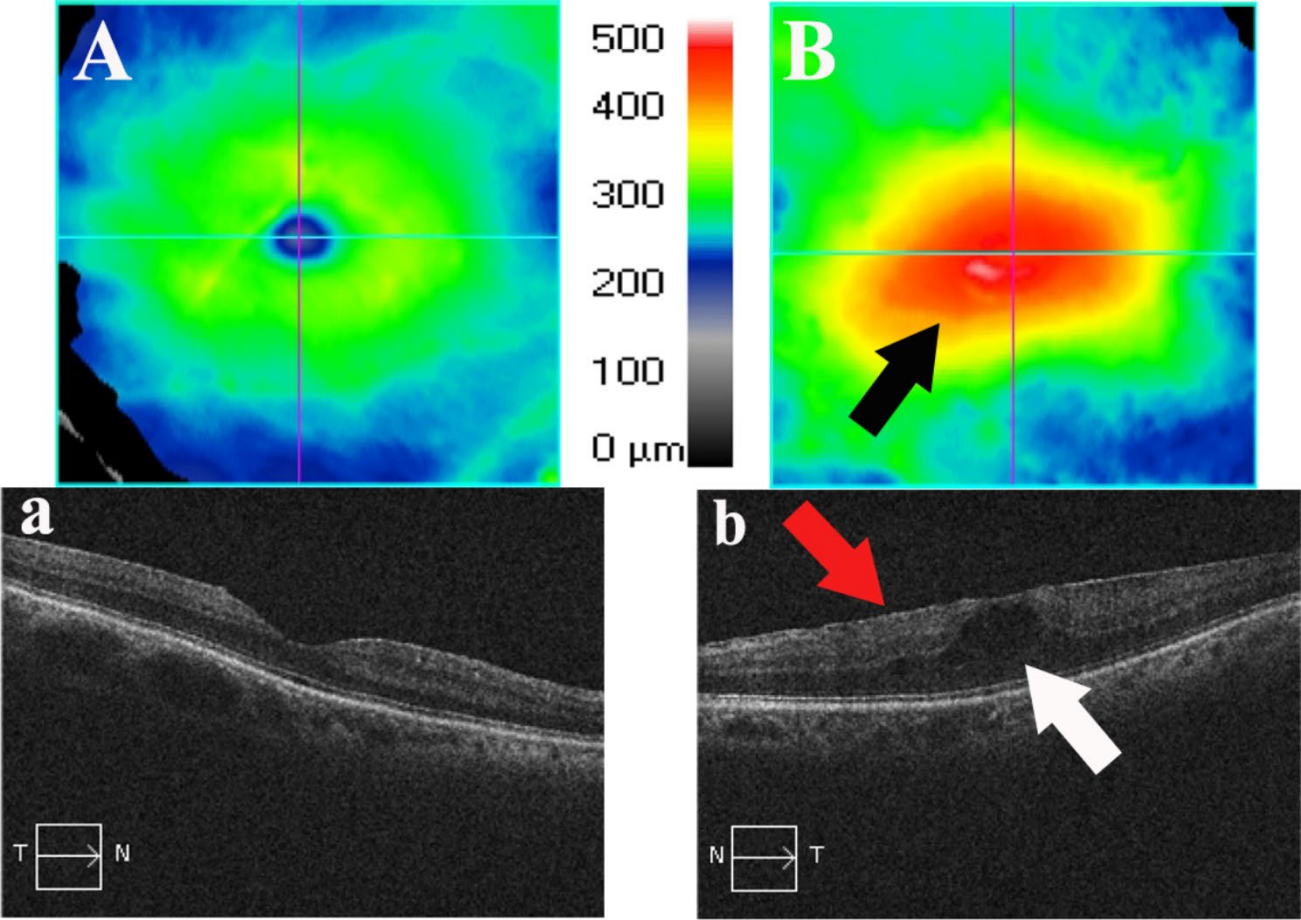
Right eye with normal macula (image **A**) and left eye with abnormal exam (image **B**). Macular edema (black and white arrow) and epiretinal membrane (red arrow) in an HTLV-1 patient (optical coherence tomography). Patient 4

Table 3 provides a more detailed description of the 12 HAU observed in this study. All HAU patients were diagnosed with HAM/TSP, and 50% had KCS. Five (41.6%, 5 of 12) subjects had anterior chamber changes, such as anterior chamber reaction, endothelial keratic precipitates, posterior synechiae, and endothelial pigment (Fig. 1). In the posterior chamber, the following changes were observed: Five (41.6%, 5 of 12) subjects had vitreous cells likely due to the sequelae of intermediate uveitis, one with an abnormality of the retinal pigment epithelium (patient 9), while four (33.3%, 4 of 12) had moderate vitreous cells characteristic of active intermediate uveitis (Fig. 2). Snowballs and papilledema were detected in one patient (8.3%1 of 12) (Fig. 3), who also had atopic and seborrheic dermatitis (patient 2), while another (8.3% 1 of 12) had macular edema and epiretinal membrane (Fig. 4) (patient 4).

Regarding the three cases of uveitis identified in the HTLV-1-uninfected group, anterior chamber changes were identified in all subjects (100%), including endothelial keratic precipitates and shallow anterior chamber; in one subject (33.3%) with active uveitis, moderate vitreous cells were observed.

**Table 3:** Characteristics of HTLV-1-infected patients with diagnosis of HTLV-associated uveitis (HAU)

Patient ID	Sex	Age (years)	HAM/TSP	HTLV-1 Diagnosis (Years)	Type of uveitis	Laterality	KCS	
**1**	F	58	**+**	< 1	Panuveitis	Unilateral	**+**	
**2**	F	29	**+**	2	Intermediate	Bilateral	**+**	
**3**	F	64	**+**	19	Intermediate	Bilateral	**-**	
**4**	F	53	**+**	11	Intermediate	Unilateral	**-**	
**5**	F	61	**+**	6	Anterior	Unilateral	**-**	
**6**	M	48	**+**	10	Intermediate	Bilateral	**-**	
**7**	M	77	**+**	16	Anterior	Bilateral	**+**	
**8**	F	49	**+**	3	Intermediate	Bilateral	**+**	
**9**	F	53	**+**	16	Intermediate	Bilateral	**-**	
**10**	F	54	**+**	3	Anterior	Unilateral	**-**	
**11**	M	59	**+**	18	Panuveitis	Bilateral	**+**	
**12**	F	69	**+**	22	Intermediate	Bilateral	**+**	

HAM/TSP: HTLV-1-associated myelopathy/tropical spastic paraparesis; KCS: keratoconjunctivitis sicca (+ present or - absent). F: female; M: male

## Discussion

The present study identified a prevalence of uveitis almost 10-fold higher in HTLV-1-infected subjects than in uninfected subjects. In addition, the characteristics of uveitis differed between HTLV-1-infected and uninfected individuals. HAU was mostly bilateral, more often localized in the intermediate chamber of the eye, and patients were asymptomatic. In contrast, uveitis in uninfected individuals was unilateral, involved the anterior chamber of the eye, and was associated with pain and visual blurring. HTLV-1 infection was the only cause associated with uveitis in the infected group, as one patient with bilateral panuveitis and Crohn’s disease and another with bilateral intermediate uveitis and HIV infection were excluded. In the HTLV-1-uninfected control group, herpes simplex and tuberculosis were found to be associated with uveitis (Table 3). Other Brazilian studies investigating ophthalmologic changes in HTLV-1 reported a lower prevalence of HAU (from 1.9 to 2.85%) than that found herein (7.14%) [19–22]. However, unlike other reports [18, 20–22], the present study was specifically designed to determine the prevalence of HAU, and a comprehensive examination procedure was performed in all patients by a single investigator trained in the diagnosis of uveitis. In addition, our sample of patients infected with HTLV-1 was larger than those involved in other studies [17, 20–22], and most of the patients investigated herein were diagnosed with HAM/TSP [19–21].

The prevalence of HAU in other endemic areas varies by geographic region. In Martinique, HAU was found in 14.5% of infected individuals [18], whereas in Japan, HAU reportedly ranges from 0.4 to 17.1%, with higher prevalence noted in the southeastern region with a higher concentration of HTLV-1 cases [14], suggesting a possible relationship between HAU development and genetic and/or environmental factors [7, 15]. This may also serve to explain differences in HAU prevalence throughout Brazil, since Bahia is an area endemic for HTLV-1 infection [3].

In addition, the route of HTLV-1 transmission may also influence the occurrence of HAU. For example, the sexual transmission of HTLV-1 has been associated with HAU development [27–29] in addition to the progression of HAM / TS [8]. Indeed, as HTLV-1 is predominantly sexually transmitted in Salvador, the capital of the state of Bahia, this fact warrants further investigation [30].

Similar to other reports, the presentation of HAU was mostly bilateral in our patients [18] and localized in the intermediate chamber [18, 19], with asymptomatic or oligosymptomatic presentations consisting of visual disturbances and floaters [18–22]. Changes in the anterior segment of the eye, vitreous cells, macular edema, epiretinal membranes, and abnormalities in retinal pigment distribution were also noted, in consonance with other reports in the literature [18–22]. However, in contrast, no retinal vasculitis was identified in the presently studied patients [19, 21, 22]. Interestingly, papilledema was observed in one case with intermediate uveitis, atopic dermatitis, and seborrheic dermatitis (Patient 2), which has not been reported by any other studies.

The pathogenesis of HAU may be explained by HTLV-1-infected T cells disrupting the blood-ocular barrier, thereby leading to intraocular inflammation [27, 31]. The production of inflammatory cytokines induced by HTLV-1-infected T cells, such as IL-1α, IL-2, IL-3, IL-8, IL-10, TFN-α and GM-CSF, produces an intraocular inflammatory environment in patients with uveitis [6]. Such inflammation leads to vitreous opacification and retinal vasculitis [32, 33], which manifests as diverse symptoms, including nebulous vision, ocular flutter, blurred vision, ocular hyperemia, ocular pain and photophobia, as well as vision loss [34]. Retinal vasculitis, known as vascular leakage, can impair retinal function and lead to visual impairment. Infiltrating cells, including HTLV-1-infected cells and inflammatory cells that can damage intraocular tissues, have been detected in the anterior chamber and vitreous in up to half of affected patients, and may lead to irreversible vision loss [34–36].

High HTLV-1 proviral load could represent a factor responsible for exacerbating the inflammatory response in HTLV-1-infected individuals, as this constitutes a biomarker for the development of inflammatory diseases, such as HAM/TSP [17, 37, 38], KCS [39] and HAU [19, 40, 41]. In Japan, HAU has been associated with the early onset of HAM/TSP [18]. Importantly, in the present study, all patients diagnosed with HAU also had HAM /TSP. Our study is limited by differences between the HTLV-1-infected and -uninfected groups, as females predominated in the infected group, and the mean age of infected subjects was significantly lower than that of the uninfected subjects. In addition, the HTLV-1-infected patients were predominantly residents of the city of Salvador, whereas the majority of the uninfected subjects resided in nearby Feira de Santana, located 100 km from Salvador. Nevertheless, the sociodemographic characteristics of the two municipalities are similar in terms of socioeconomic and ethnic characteristics [42].

In conclusion, our results indicate a higher prevalence of uveitis was in HTLV-1-infected subjects than in uninfected controls. HAU patients are usually asymptomatic or oligosymptomatic. Visual disturbances may go unnoticed and can potentially lead to permanent visual damage. The findings herein serve to confirm the importance of regular ophthalmologic examinations in HTL-1-infected patients in order to achieve early diagnosis and properly treat uveitis.

## Data Availability

The raw data supporting the conclusions of this article will be made available by the authors, without undue reservation.
